# Cognitive Function of Children and Adolescents With Congenital Adrenal Hyperplasia: Importance of Early Diagnosis

**DOI:** 10.1210/clinem/dgaa016

**Published:** 2020-01-12

**Authors:** Valeria Messina, Leif Karlsson, Tatja Hirvikoski, Anna Nordenström, Svetlana Lajic

**Affiliations:** 1 Department of Women’s and Children’s Health, Karolinska Institutet, Pediatric Endocrinology Unit, Karolinska University Hospital, Stockholm, Sweden; 2 Department of Women’s and Children’s Health, Pediatric Neuropsychiatry Unit, Center for Neurodevelopmental Disorders at Karolinska Institutet (KIND), Karolinska Institutet, Stockholm, Sweden; 3 Unit for Habilitation & Health, Stockholm County Council, Stockholm, Sweden

**Keywords:** cognitive functions, congenital adrenal hyperplasia, glucocorticoids, dexamethasone, prenatal treatment

## Abstract

**Context:**

Patients with classic congenital adrenal hyperplasia (CAH) are treated postnatally with lifelong glucocorticoid (GC) replacement therapy. Previous results on general cognitive ability in individuals with CAH have been conflicting.

**Objective:**

To evaluate long-term cognitive effects of GC replacement therapy and the impact of early diagnosis in children with CAH.

**Design and Setting:**

Observational study with patients from a single research institute.

**Patients:**

32 children with CAH (mean age 11.5 years) identified through the Swedish national neonatal screening program for CAH and 52 matched population controls (mean age 10.7 years). Eleven (6 female) children with CAH who were treated prenatally with dexamethasone (DEX), (CAH-DEX) (mean age 11.7 years).

**Intervention:**

GC replacement therapy, neonatal screening for CAH.

**Measures:**

Cognitive abilities assessed with standardized neuropsychological tests (Wechsler scales, Span Board Test, Stroop Interference Test, NEPSY list learning).

**Results:**

Children with CAH (not prenatally treated) performed equally well as population controls on a series of tests assessing general intellectual ability and executive functions. No significant differences were observed in cognitive performance between patients with different genotypes (null, non-null). Patients with salt-wasting CAH performed poorer than patients with simple virilizing CAH in a test assessing visuo-spatial working memory (*P* = 0.039), although the performance was within the normal range for the population. Prenatally DEX-treated girls with CAH had lower verbal intellectual ability compared with CAH girls not exposed to prenatal treatment (*P* = 0.037).

**Conclusion:**

Children and adolescents with CAH who were diagnosed early via a neonatal screening program and treated with hydrocortisone had normal psychometric intelligence and executive functions.

Congenital adrenal hyperplasia (CAH) is most commonly caused by mutations in the *CYP21A2* gene (resulting in 21-hydroxylase deficiency, 21OHD) and has a prevalence of 1:15.000 in most populations (1). Patients with 21OHD have an impaired synthesis of cortisol and, in more severe cases, an impaired synthesis of aldosterone. Traditionally, 21OHD has been classified into the classical forms, salt-wasting (SW) CAH (with aldosterone deficiency) and simple-virilizing (SV) CAH, or the milder, late-onset non-classic form (NC CAH).

The synthetic glucocorticoid (GC) dexamethasone (DEX) has been administered to pregnant mothers who have previously given birth to a child with severe CAH to reduce virilization of external genitalia in the affected female fetus (2). Although effective in ameliorating the genital virilization, the treatment is controversial because of uncertainties regarding long-term safety (2,3). Adding to the concern is the fact that the doses of DEX that the fetus is exposed to are estimated to be 60 times the normal fetal cortisol level (4).

Postnatally, patients with classic CAH are treated with lifelong GC replacement therapy (e.g., hydrocortisone [HC] or prednisolone) but, because of difficulties in mimicking the circadian cortisol rhythm, the GC concentration can be suboptimal, with a risk of both over- and undertreatment (5). In addition, patients with classic CAH are at risk of adrenal crisis with salt loss and hypoglycemia that may negatively impact cognitive performance (6).

The glucocorticoid and the mineralocorticoid receptors are highly expressed in the hippocampus, amygdala, and prefrontal cortex (7, 8). These areas, important for executive functioning, emotional regulation, and memory, are vulnerable to high doses of GCs (9). Therefore, it seems reasonable to infer that the combination of long-term GC replacement therapy and prenatal treatment with DEX might affect cognitive and affective functions in patients with CAH.

Previous results on general cognitive ability in patients with CAH have been conflicting, and the majority of the studies have focused on intelligence in adult patients. Some studies have reported normal intelligence in both women and men with CAH (10), whereas others have noted impaired intelligence (11, 12) and memory deficit (13) in children and adults with CAH. In an epidemiological study, women with SW CAH had completed primary education less often (OR 0.3) compared with population controls (14). A recent investigation demonstrated that patients with CAH have impaired spatial perception and diminished quantitative abilities, most probably due to altered executive functioning (15–17). On the contrary, others have reported superior performance on spatial tests in women with CAH (18–20).

In addition to the effects of postnatal GC, an excess of prenatal androgens may be associated with permanent changes in brain structures, organization, or function (21).

Prenatal treatment with DEX may also affect cognitive functions. A recent report indicated poorer cognitive abilities in DEX-treated women with CAH compared with women with CAH who received no prenatal DEX therapy (17).

Neonatal screening for CAH is performed in an increasing number of countries all over the world (22, 23). Sweden has had a national neonatal screening for CAH since 1986 (24) and it has been shown to be effective in preventing adrenal crisis and neonatal death in infants with SW CAH (25). The long-term effects on cognition of neonatal crises are difficult to assess but we may hypothesize that early diagnosis and avoiding hypocortisolism and salt crises in the neonatal period leads to a more favorable cognitive development.

In the current study, we therefore investigated the cognitive outcome of a Swedish cohort of children and adolescences with CAH (age 7–17 years) diagnosed through the national neonatal screening program. We compared their performance with the performance of healthy children without CAH from the general population. In addition, since there are important differences in the clinical outcome for various genotype subgroups in CAH we examined the impact of CAH genotype and phenotype on cognition (26, 27).

We also present data on a small cohort of patients with CAH (n = 11) who were treated prenatally with DEX. Thus, this study aimed to examine the effects of early diagnosis and GC treatment on cognitive function in children and adolescents with CAH.

## Materials and Methods

### Subjects

The study is part of a longitudinal cohort study (PREDEX) investigating short- and long-term effects of prenatal treatment with DEX in fetuses at risk for CAH and the effects of postnatal treatment with GCs in individuals with CAH (17, 28-30). Here we focus on the cognitive function of children and adolescents with CAH and we also observe a small group of patients with CAH who were treated with DEX prenatally (CAH-DEX group; boys during the first trimester and girls during the entire gestational period, starting at about gestational week 7. Children/adolescents with CAH resided in cities throughout Sweden while the control group (C) was randomly selected from the population in Stockholm County through the Swedish Population Registry and matched for sex and age. Each patient was matched with 1 to 2 selected controls. Subjects were tested during years 2002–2004 and 2013–2016. Parents gave their written informed consent before inclusion and the study was approved by the Regional Ethics Committee in Stockholm. The study participation rate for the CAH cohort was 81.0% and for the population controls 55.0%.

In total, the CAH sample comprised 43 children and adolescents with CAH (age range 7–17 years; 23 girls, 20 boys; including 1 sibling pair). Of these 43 children/adolescents, 11 (24%) (5 boys, 6 girls) had been prenatally treated with DEX.

All patients with CAH, except 1 (not born in Sweden), were identified through the Swedish national neonatal screening program and diagnosis was confirmed with genotyping of the *CYP21A2 gene*. Thirty patients had SW CAH, 12 had SV CAH and 1 patient had NC CAH (though this child received DEX prenatally due to technical problems with the prenatal genotyping). Among the patients with CAH, 12 had a null genotype (completely abolished 21-hydroxylase activity) and 30 had a non-null genotype (genotypes with some residual 21-hydroxylase activity). The genotype was not known in 1 patient.

In 25 of 30 patients with the SW phenotype we could identify the basal sodium levels at the time of diagnosis and only 2 patients exhibited a sodium level below 131 mmol/L. All patients were treated with HC at the time of cognitive testing (n = 42; mean dose 12.3 ± 2.9 mg/m^2^/day). The mean fludrocortisone dose at the time of testing was 97.9 ± 41.7 μg/day (n = 35).

The control group comprised 52 individuals (age range 7–17 years; 27 girls, 25 boys) including 3 who were siblings of 3 patients with CAH and who opted to participate in the study.

Demographic data for the separate groups are summarized in Tables 1 and 2. Age and parental education did not differ between the groups (all *P* > 0.05). Length and weight at birth were converted into z-scores according to Swedish population-based longitudinal reference values (31). Patients with CAH (not prenatally treated) were on average larger than population controls (birth length, *P* = 0.027; birth weight, *P* = 0.038) (corrected for gestational age) (Table 1). CAH-DEX participants did not differ in birth length or weight compared with CAH children not treated with DEX and matched for sex and age, (Table 2). CAH-DEX boys were treated with a higher dose of HC compared with boys with CAH not prenatally treated with DEX (*P =* 0.002) at the time of cognitive testing, (Table 2).

**Table 1.** Demographic Data for the Entire Study Population

	*Female*			*Male*			
	CAH	CAH-DEX	C	CAH	CAH-DEX	C	*P* Value (CAH) CAH vs C
**Number of patients**	17	6	27	15	5	25	
**Age, mean** (**SD)**	11.2 (3.2)	11.8 (3.8)	10.6 (2.5)	11.7 (2.6)	11.5 (0.9)	11.0 (2.1)	0.215
**HC (mg/m** ^**2**^ **), mean** (**SD)**	12.5 (3.4)	12.4 (3.6)	N/A	11.3 (1.5)	14.6 (2.5)	N/A	
**Fludrocortisone (μg), mean (SD)**	110.7^3^ (47.7)	75.0^4^ (25.0)		96.4^5^ (39.0)	83.3^6^ (28.9)		
**SES/Parental academic exam, n (%)** ^**1**^	43.8	50.0	84	76.9	40.0	52.0	0.340
**Birth length (z-score)**	0.5 (2.1)	–0.7 (1.4)	–0.0 (1.3)	0.5 (1.3)	–0.2 (1.6)	–0.7 (1.4)	0.027
**Birth length (cm)** ^**2**^ **, mean (SD)**	50.3 (3.9)	48.1 (2.2)	49.3 3.6)	52.1 (1.8)	51.6 (3.2)	49.6 (3.0)	
**Birth weight (z-score)**	0.1 (0.5)	–0.2 (0.5)	–0.1 (0.5)	0.1 (0.3)	–0.1 (0.5)	–0.2 (0.5)	0.038
**Birth weight (g)** ^**2**^ **, mean (SD)**	3488.5 (797.6)	2977.5 (653.1)	3283.0 (681.0)	3748.1 (363.9)	3663.0 (752.0)	3358.1 (745.1)	
**Gestational week, mean (SD)**	39.1 (2.3)	38.1 (2.2)	39.6 (2.0)	39.8 (2.0)	40.3 (1.1)	39.5 (2.3)	0.683

Patients with CAH not treated prenatally with DEX (CAH), patients with CAH treated prenatally with DEX (CAH-DEX) and population controls (C). Children with CAH (not prenatally treated) were born larger than population controls. Statistical analyses for differences between groups were calculated using Fisher’s exact test (two-sided).

Abbreviations: CAH, congenital adrenal hyperplasia; CAH-DEX, CAH who were treated prenatally with DEX; DEX, dexamethasone; HC, hydrocortisone dose; N/A, not applicable; SD, standard deviation; SES, socioeconomic status.

^1^SES estimated as parental education at university level (%).

^2^Values not adjusted for gestational age.

^3^n = 14; ^4^n = 5; ^5^n = 14; ^6^n = 3.

**Table 2.** Demographic Data for the CAH-DEX Cohort (n = 11) and for the CAH Subgroup (n = 21)

	*Female*		*Male*			
	CAH	CAH-DEX	CAH	CAH-DEX	*P* (DEX) CAH-DEX vs CAH (f)	*P* (DEX) CAH-DEX vs CAH (m)
**Number of patients**	11	6	10	5		
**Age, mean** (**SD)**	11.1 (3.6)	11.8 (3.8)	11.6 (1.5)	11.5 (0.9)	0.717	0.869
**HC (mg/m** ^**2**^ **) , mean** (**SD)**	12.4 (4.1)	12.4 (3.6)	10.9 (1.4)	14.6 (2.5)	0.967	0.002
**Fludrocortisone (μg), mean (SD)**	128.6^3^ (54.8)	75.0^4^ (25.0)	102.8^5^ (47.5)	83.3^6^ (28.9)	0.071	0.526
**SES/Parental academic exam, n (%)** ^**1**^	50.0	50.0	75.0	40.0	1.000	0.241
**Birth length (z-score)**	0.3 (2.4)	–0.7 (1.5)	0.9 (1.5)	–0.2 (1.6)	0.349	0.215
**Birth length (cm)** ^**2**^ **, mean (SD)**	50.2 (4.6)	48.1 (2.2)	52.5 (2.0)	51.6 (3.2)		
**Birth weight (z-score)**	0.1 (0.6)	–0.2 (0.4)	0.1 (0.4)	–0.1 (0.5)	0.337	0.424
**Birth weight (g)** ^**2**^ **, mean (SD)**	3502.6 (930.1)	2977.5 (653.1)	3825.2 (274.8)	3663.0 (752.0)		
**Gestational week (SD)**	39.4 (2.4)	38.1 (2.2)	40.1 (1.7)	40.3 (1.1)	0.286	0.816

The CAH subgroup was matched for sex and age with the CAH-DEX patients and used as a control group in the between group analyses. CAH-DEX boys were treated with a higher dose of HC at the time of testing, compared with CAH boys not prenatally treated.

Abbreviations: CAH, congenital adrenal hyperplasia; CAH-DEX, CAH who were treated prenatally with DEX; DEX, dexamethasone; HC, hydrocortisone dose; N/A, not applicable; SD, standard deviation; SES, socioeconomic status.

^1^SES estimated as parental education at university level (%).

^2^Values not adjusted for gestational age.

^3^n = 7; ^4^n = 5; ^5^n = 9; ^6^n = 3

Statistical analyses for differences between groups were calculated using Fisher’s exact test (two-sided).

The biochemical control was checked at the time of cognitive testing in order to ensure that there were no outliers with a poor metabolic control which could affect the ability to perform the test session. The blood 17-hydroxyprogesterone (B-17OHP) levels were evaluated using at home capillary blood sampling prior to each given dose of HC (in total 4 measurements per 24 hours) for a time interval of 0 to 3 months before or after the time point for cognitive testing (original files can be made available upon request). In general, patients with CAH in Sweden are followed on a routine basis with this type of 17OHP measurement once every 3 months. In general, the patients included in the study had a good metabolic control. Two patients had elevated 17OHP levels in the morning but still within a range that made them eligible to be included in the study (B-17OHP < 300 nmol/L).

### Outcome measures of cognitive functions

This study focused on general intellectual ability, executive functions, and learning and memory, which were evaluated using standardized neuropsychological tests. Trained psychologists assessed all participants. The study group status was not blinded, but the psychologists were not explicitly informed about specific data such as type of prenatal DEX exposure or CAH phenotype. Each participant was tested individually in a quiet environment. The neuropsychological tests were administered in a fixed order following a research protocol and were completed in 1 session by the same psychologist with 1 break of 15 minutes to avoid systematic effect of fatigue. All the tests were completed in approximately 1.5 hours.

### General intellectual ability

General intellectual ability (estimation of psychometric intelligence) was measured with 2 subtests from the Wechsler Intelligence Scale for Children-III (WISC-III): the Block Design subtest for estimating nonverbal (performance) intelligence and the Vocabulary subtest for estimating verbal intelligence (32). There is a good correlation between Vocabulary and the Verbal comprehension index (86%) and between Block Design and the Perceptual organization index (77%) (32).

### Executive functions

The Digit Span subtest from the WISC-III was selected to measure verbal working memory (32, 33), and the Span Board subtest from the WMS-III was applied to assess visual-spatial working memory (34). The participants were administered the Coding subtest of the WISC-III to measure speed in information processing (32). The Stroop Interference test was used to evaluate the ability to inhibit an overlearned response (35).

### Learning and memory

The list learning subtest of the NEPSY (a developmental neuropsychological assessment) was administered to measure learning and long-term memory (36).

### Statistical analyses

The raw scores of the WISC-III subtests were transformed into scaled scores based on age-specific Swedish norms in order to facilitate comparison with the population mean (population norm M = 10, SD = 3) (32). Although normed in Sweden, the Swedish version of the NEPSY test does not include the scaled scores. Thus, to enable comparison between the different subtests we transformed the NEPSY raw scores into scaled scores (M = 10, SD = 3) according to the American manual (36). The raw scores of the Span Board subtest were transformed into T-scores (population norm M = 50, SD = 10) using the norm tables of the Wechsler Nonverbal Scale of Ability (WNV) (34). In addition, the results of the Stroop Interference test were transformed into T-scores according to the American norms (35). All data are thus age-adjusted according to age-specific norms.

Two-way analyses of variance (ANOVAs) with sex (male vs female) and group (CAH vs control) as the independent variables were used to compare CAH patients not prenatally treated with DEX with general population controls. Interactions (*P* < 0.05) between sex and group were followed up by separate post hoc comparisons between patients and controls of the same sex.

In addition, we performed Mann-Whitney *U* tests to compare cognitive performance in children with CAH who were treated prenatally with DEX with those with CAH not prenatally DEX-treated. The Mann-Whitney *U* tests were performed because of the small sample size of the CAH-DEX group and because the data from the Vocabulary subtest did not follow a normal distribution. The CAH-DEX group was matched for sex and age with a subgroup of the CAH group.

In all analysis we also controlled for parental education. We also compared test performance between children/adolescents with CAH (not prenatally treated) with different phenotypes (SW, SV) and genotype groups (null, non-null) using one-way ANOVAs. For post hoc tests between phenotypes, we only analyzed boys, given that the SW group included only 2 females.

The impact of the GC replacement dose (mg/m^2^/day) at the time of testing of cognitive performance was analyzed using Pearson’s bivariate correlation coefficients.

Effect sizes were expressed as Cohen’s *d* and classified as large when *d ≥ *0.80, medium when *d ≥ *0.50 and small when *d ≥ *0.20 (37). For comparisons between groups, a two-tailed alpha level of *P* < 0.05 was used. To avoid missing clinically relevant effects, we did not correct for multiple comparisons and all analyses were planned a priori. SPSS version 23 (IBM, Armonk, NY) was used for statistical analysis.

## Results

Here, we present the cognitive outcomes of children and adolescents with CAH compared with a control group from the general population. Data on the cognitive performance are also presented in a small group of prenatally DEX-treated patients with CAH compared with those with CAH who did not receive such treatment.

### Estimates of general intellectual ability

No statistically significant differences in estimates of general intellectual ability were observed between the CAH cohort and the controls (Table 3). In addition, there were no significant interactions between sex and diagnostic status (*P* > 0.05) (Table 3).

**Table 3.** Results of the Neuropsychological Tests Comparing CAH Patients With Population Controls (means ± 1 SD)

	*Female*		*Male*		*[CAH]*			*[CAH × Sex]*	
	CAH (n = 17)	Control (n = 27)	CAH (n = 15)	Control (n = 25)	Effect size (Cohen’s d)	F statistics	*P*	F statistics	*P*
**General intellectual ability (estimated IQ)**									
WISC-III Block Design (S)	10.7 (3.5)	11.6 (2.8)	11.7 (2.9)	11.0 (3.1)	–0.06	0.5	0.819	0.79	0.377
WISC-III Vocabulary (S)	11.3 (2.7)	11.1 (2.3)	11.9 (1.6)	10.4 (2.1)	0.52	2.38	0.127	3.10	0.082
**Executive functions**									
WISC-III Coding (S)	11.6 (2.8)	12.3 (3.2)	10.6 (2.1)	9.4 (2.5)	0.11	0.01	0.926	1.60	0.209
WISC-III Digit Span (S)	9.2 (2.7)	10.7 (3.3)	11.0 (3.1)	10.6 (3.2)	–0.27	0.44	0.508	2.21	0.141
Span Board Forward (T)	51.2 (10.5)	53.2 (10.0)	55.0 (10.4)	50.7 (10.6)	0.13	0.32	0.574	0.61	0.438
Span Board Backward (T)	56.0 (8.9)	56.7 (8.8)	60.2 (7.9)	54.9 (8.0)	0.36	2.22	0.141	2.53	0.116
Stroop interference (T)	54.8 (4.1)	52.4 (4.9)	52.5 (8.2)	51.0 (3.5)	0.50	0.92	0.340	1.10	0.297
**Learning and long-term memory**									
NEPSY List Learning (S)	12.4 (2.1)	11.0 (3.2)	11.5 (2.3)	10.7 (2.7)	0.61	1.53	0.221	0.45	0.502

Effect sizes (Cohen’s *d*) and *P* values are shown for all findings. Positive effect sizes represent higher scores in the CAH cohort, whereas negative effect sizes represent higher scores in the control group (C). No statistically significant differences were observed between the groups.

Scaled scores: population mean = 10 (SD = 3); T-scores: population mean = 50 (SD = 10).

Abbreviations: CAH, congenital adrenal hyperplasia; NEPSY, Developmental Neuropsychological Assessment; S, scaled score; T, T-score; WISC-III, Wechsler Intelligence Scale for Children-III.

We did not observe any significant differences between phenotypes (SW vs SV, Table 4) or genotypes (null vs non-null) in any measure of cognitive ability (all *P >* 0.05). There were no significant correlations between the HC dose at the time of cognitive testing and measures of general intellectual ability (all *P >* 0.05).

**Table 4.** Scores on the Neuropsychological Tests (mean ± 1 SD) for Patients With SW CAH vs Patients With SV CAH

	SW (n = 20)	SV (n = 12)	Cohen’s *d*	F statistics	*P*
**General cognitive ability (estimated IQ)**					
WISC-III Block Design (S)	10.4 (4.0)	12.8 (1.1)	-1.25	3.97	0.058
WISC-III Vocabulary (S)	11.0 (2.6)	12.4 (1.3)	-0.57	3.50	0.073
**Executive functions**					
WISC-III Coding (S)	10.9 (3.1)	11.2 (1.8)	0.02	0.04	0.855
WISC-III Digit Span (S)	9.5 (3.3)	10.4 (2.4)	-0.20	0.67	0.420
Span Board Forward (T)	50.0 (11.0)	58.5 (8.9)	-1.20	4.72	**0.039**
Span Board Backward (T)	57.5 (6.7)	62.5 (7.9)	-1.03	4.01	0.056
Stroop interference (T)	53.2 (5.8)	52.8 (7.5)	0.01	0.02	0.898
**Learning and long-term memory**					
NEPSY List Learning (S)	12.2 (1.9)	11.6 (2.8)	0.25	0.31	0.583

Effect sizes (Cohen’s *d*), F statistics and p-values are shown for the entire population (data not split by sex). Positive effect sizes represent higher scores in the SW group, whereas negative effect sizes represent higher scores in the SV group. Patients with SW CAH performed poorer than patients with SV CAH on Span Board Forward.

Scaled scores: population mean = 10 (SD = 3); T-scores: population mean = 50 (SD = 10).

Abbreviations: CAH, congenital adrenal hyperplasia; NEPSY, Developmental Neuropsychological Assessment; SW, salt-wasting; SV, simple-virilizing; S, scaled score; T, T-score; WISC-III, Wechsler Intelligence Scale for Children-III.

### Executive functions

No significant group differences were found between the patients with CAH and the controls on subtests assessing executive functions (all *P >* 0.05) (Table 3). There were no significant interactions between sex and diagnostic status (all *P >* 0.05) (Table 3).

When comparing the SW and SV groups within the CAH cohort, the patients with SW CAH had significantly lower test scores than the patients with SV CAH in the Span Board forward subtest (F(1,25) = 4.72, *P =* 0.039, *d = *−1.20) (Table 4). However, separate post hoc analyses comparing only boys did not reach statistical significance (F (1,14) = 0.21, *P =* 0.650). We did not find any significant difference in executive functions between the patients who had different genotypes (null vs non-null). There were no significant correlations between the HC dose at the time of cognitive testing and measures of executive functions (all *P >* 0.05).

### Learning and memory

The CAH cohort performed equally well as the controls in the NEPSY List Learning subtest (*P >* 0.05, Table 3). There were no significant interactions between sex and diagnostic status (*P >* 0.05) (Table 3). No significant effects were identified for the HC dose at the time of cognitive testing (*P >* 0.05), the phenotype (SW vs SV) (Table 4), or the genotype (null vs non-null).

### Effects of prenatal dexamethasone treatment

Girls with CAH who had been treated prenatally with DEX showed lower scores on the Vocabulary subtest of the WISC-III (*U = 12*.00, *P =* 0.037, *d = −*1.75) (Table 5).

**Table 5.** Results of Neuropsychological Tests Comparing Female and Male CAH Patients Treated Prenatally With DEX (CAH-DEX) with Female and Male CAH Patients Not Treated Prenatally With DEX (CAH)

	Females					Males				
	CAH-DEX (n = 6)	CAH (n = 11)	Cohen’s *d*	Mann Whitney U test		CAH-DEX (n = 5)	CAH (n = 10)	Cohen’s *d*	Mann Whitney U test	
				*U*	*p*				*U*	*p*
**General intellectual ability (estimated IQ)**										
WISC-III Block Design (S)	9.2 (3.1)	9.9 (3.0)	−0.29	28.00	0.660	11.0 (3.4)	11.0 (3.2)	0.00	21.00	0.898
WISC-III Vocabulary (S)	8.0 (2.7)	11.4 (2.8)	−1.75	12.00	**0.037**	11.0 (1.6)	12.1 (2.0)	−0.86	15.00	0.254
**Executive functions**										
WISC-III Coding (S)	10.0 (3.0)	12.1 (3.3)	−0.94	18.00	0.220	11.6 (2.1)	11.0 (2.2)	−0.40	22.00	0.768
WISC-III Digit Span (S)	9.3 (1.9)	9.4 (2.5)	−0.07	32.50	0.961	8.6 (3.6)	10.5 (2.1)	−0.90	18.00	0.440
Span Board Forward (T)	47.8 (7.5)	46.5 (9.0)	0.22	25.50	0.462	49.7 (17.4)	56.8 (10.1)	−0.70	17.00	0.733
Span Board Backward (T)	52.8 (3.4)	53.4 (8.6)	−0.13	28.00	0.660	49.5 (7.7)	60.4 (7.4)	−2.04	4.00	0.024
Stroop interference (T)	56.0 (6.4)	55.3 (4.3)	0.18	24.50	0.953	54.6 (3.3)	54.8 (9.0)	−0.04	23.00	0.859
**Learning and long-term memory**										
NEPSY List Learning (S)	10.7 (3.6)	12.8 (1.8)	−1.05	20.50	0.216	12.0 (2.0)	11.2 (1.8)	0.57	16.50	0.438

Scaled scores: population mean = 10 (SD = 3); T-scores: population mean = 50 (SD = 10).

Abbreviations: CAH, congenital adrenal hyperplasia; CAH-DEX, CAH who were treated prenatally with DEX; DEX, dexamethasone; NEPSY, Developmental Neuropsychological Assessment; S, scaled score; T, T-score; WISC–III, Wechsler Intelligence Scale for Children-III.

Boys in the CAH-DEX group, who were treated prenatally with DEX during the first trimester of fetal life, showed scores within the normal range for the population and did not differ from boys with CAH who were not treated with DEX (all *P >* 0.05) (Table 5).

## Discussion

In this study we compared cognitive functions in Swedish children and adolescents with CAH who were not prenatally treated with DEX with general population controls. We also compared the cognitive performance in a small subgroup of patients with CAH who had been treated prenatally with DEX (i.e., the CAH-DEX group) with non-DEX-treated patients with CAH.

The patients with CAH showed normal general intelligence and executive functions when compared with controls from the general population. We could not find any differences in cognitive outcome between patients with different genotypes (null or non-null); nor were we able to detect any association between the current dose of HC at the time of testing and cognitive outcome. However, the patients with the SW phenotype performed poorer than those with SV CAH in a measure of visuo-spatial working memory (Span Board Forward), even if all the scores were within the average range for the population. Because our cohort has only 2 SV females with CAH we cannot conclude if there is an interaction with sex.

Previous studies investigating general intellectual ability and working memory in patients with CAH are contradictory (largely because samples vary, and the methods used are different). Our results are consistent with some (10) but not all earlier findings (15, 16, 38). In the study by Brown and colleagues, children with CAH had an impaired verbal working memory already at a young age (7–11 years) (15) and this was also identified during adolescence and at adult age (16). Hampson and Rovet, identified a reduced Full Scale IQ in female patients with SW CAH and the patients included in the study did not undergo neonatal screening (38).

In the UK and in many other countries worldwide, neonates are not screened at birth for CAH as they are in Sweden. Hence, an earlier diagnosis and treatment in our screening-detected cohort could account for equal performance in children with CAH compared to healthy peers from the general population. Our CAH cohort was detected through the Swedish national neonatal screening program, which prevents adrenal crisis and early infant death in cases with classic CAH (25). The present data on cognitive outcome thus reinforces the positive value of the Swedish neonatal screening program.

In our previous study of adults with CAH (female mean age 25 years, male mean age 23 years) we observed some negative effects on cognitive function, indicating that a cumulative dose of GCs over the life course may be linked to an increased loss of cognitive abilities (17).The current cohort of children and adolescents with CAH (7–17 years) received HC as GC replacement, whereas the adults in our previous study were treated with HC or prednisolone, or a combination of both. At present, it is not known whether the long-term effects on cognitive performance differ between the different GCs in the context of replacement therapy. Nevertheless, it cannot be ruled out that management issues during the transition from pediatric to adult care are of importance. Multiple salt-losing crises (6) and hypoglycemia (39) will also affect cognition. Therefore, new medical treatments, transition protocols, and access to multidisciplinary teams must be taken into account to improve the long-term outcomes in adults with CAH.

In the present study we observed differences in cognitive performance between the SW and SV CAH groups, differences that contrast with our previous study in adults with CAH (17). However, some other reports indicate that patients with the SW phenotype tend to have poorer cognitive performance (12). This is not surprising since a more severe phenotype may predispose the individual to more salt-losing crises and include a higher GC load that may affect areas in the brain that are involved in working memory processes. As previously mentioned, we could not form conclusions whether there was an interaction with sex because there were only 2 girls with SV CAH in this study.

A dose-dependent effect on associative learning and long-term memory has been reported in asthmatic children and adults treated with GCs (40, 41), denoting an effect of corticosteroids on the processes involved in memory formation (42). In the present study we did not observe a different performance in children and adolescents with CAH in a subtest assessing learning and long-term memory compared to controls.

In summary, good medical treatment with HC, a good adherence to therapy, and a rapid initiation of treatment facilitated by nationwide neonatal screening may all contribute to the positive effects on cognition that we see in the children and adolescents with CAH in Sweden.

An important component in the management of CAH is the prenatal treatment with DEX. For many years, our group has studied the long-term effects of prenatal treatment with DEX on cognition and behavior. In the present study, we report on a small and unique group of patients (6 females, 5 males) with CAH prenatally treated with DEX. Our findings may indicate a poorer performance on a subtest assessing verbal intelligence in DEX treated girls—although due to poor statistical power we are not able to draw any formal conclusion and the performance was, although towards the lower part of the reference range, still within the normal range for the population. However, our data are concordant with a previous study from Meyer-Bahlburg et al that indicated slower cognitive processing in long-term DEX-treated girls compared with short-term DEX-treated girls (43).

Consequently, our findings on children with CAH and on long-term prenatal DEX exposure are not definitive and need replications in larger samples.

In conclusion, our results suggest that children and adolescents diagnosed through the neonatal screening program for CAH in Sweden have normal cognitive abilities when compared with healthy controls from the general population. This emphasizes the importance of the neonatal screening program for CAH. Nevertheless, it cannot be ruled out that episodes of hyponatremia or hypoglycemia can affect cognition later in life Optimal treatment with GCs (HC) appears to be an important and likely explanation for the similar test performance in the children and adolescents with CAH and the controls. Further studies are needed to evaluate cognition in patients with a different treatment regimen. Additional retrospective and longitudinal studies are necessary to evaluate the risks and benefits of prenatal DEX treatment.

## Abbreviations

17OHP: 17-hydroxyprogesterone
21OHD: 21-hydroxylase deficiency
ANOVA: analysis of variance
B-17OHP: blood 17-hydroxyprogesterone
C: control
CAH: congenital adrenal hyperplasia
CAH-DEX: CAH who were treated prenatally with DEX
DEX: dexamethasone
GC: glucocorticoid
HC: hydrocortisone
NC CAH: non-classic congenital adrenal hyperplasia
SW: salt-wasting
SV: simple-virilizing
WISC-III: Wechsler Intelligence Scale for Children-III

## Additional Information

***Disclosure Summary:*** The authors have nothing to disclose.

***Data Availability:*** The datasets generated during and/or analyzed during the current study are not publicly available but are available from the corresponding author on reasonable request.
